# Association of heavy metal with sperm parameters and sex hormones: A systematic review and meta-analysis of observational studies

**DOI:** 10.1016/j.eurox.2026.100505

**Published:** 2026-10-16

**Authors:** Saba Goodarzi, Arman Shafiee, Pegah Rashidian, Reza Ghoreshi, Shirin Djalalinia, Zahra Nouri Ghonbalani, Razieh Lotfi, Zohreh Mahmoodi, Mostafa Qorbani

**Affiliations:** a Non-Communicable Diseases Research Center, Alborz University of Medical Sciences, Karaj, Iran; b Student Research Committee, Alborz University of Medical Sciences, Karaj, Iran; c Vali-e-Asr Reproductive Health Research Center, Family Health Research Institute, University of Medical Sciences, Tehran, Iran; d Non-Communicable Diseases Research Center, Endocrinology and Metabolism Population Sciences Institute, Tehran University of Medical Sciences, Tehran, Iran; e Social Determinants of Health Research Center, Alborz University of Medical Sciences, Karaj, Iran

**Keywords:** Heavy metal, Trace element, Sperm analysis, Sex hormones, Infertility, Reproductive health, Cadmium, Lead

## Abstract

**Background:**

Findings regarding the reproductive toxicity effect of heavy metals on sperm parameters and sex hormones level remain inconsistent. This study systematically reviewed and pooled the existing literature to assess the associations of heavy metal levels with sperm parameters and sex hormones.

**Methods:**

In this systematic review, international databases including PubMed, Scopus, and Web of Science were searched until 31 March 2025 according to PRISMA guidelines. Observational studies assessing heavy metal concentrations in biological samples and their associations with sperm parameters or sex hormone levels were included. The chi-square-based Q test and I-squared statistics were used to assess the heterogeneity, and pooled estimates were obtained using a random-effects meta-analysis model.

**Results:**

Eighteen studies involving 4041 participants were included. Qualitative synthesis revealed heterogeneous findings across heavy metals and sperm parameters, with significant findings reported mainly for Cd and Pb, while most associations with reproductive hormones were non-significant. Meta-analysis demonstrated a significant inverse association between seminal Cd and sperm motility (pooled Fisher’s z = -0.17, 95% CI: −0.30 to −0.04; I² = 0%), whereas no significant pooled associations were observed for seminal Pb.

**Conclusions:**

The current evidence does not demonstrate consistent associations between heavy metal exposure and male reproductive parameters; however, limited significant inverse correlations were identified between seminal Cd and sperm motility. Evidence regarding reproductive hormones was limited and largely inconclusive, highlighting the need for larger, well-designed prospective studies.

## Introduction

Infertility is a global public health concern, affecting approximately 10–15% of reproductive-aged couples worldwide, with complex etiologies that include genetic, hormonal, lifestyle, and environmental factors [1], [2]. Testicular dysfunction, endocrine abnormalities, genetic factors, lifestyle-related exposures, and environmental or occupational gonadotoxicants contribute to approximately 30–50% of infertility cases [3].

Heavy metals are ubiquitous in the environment due to industrial emissions, agricultural practices, contaminated food and water, and occupational exposure [4]. These metals are not biodegradable and tend to accumulate in biological tissues over time, exerting toxic effects even at low concentrations. Several studies have shown that heavy metals can disrupt endocrine signaling, induce oxidative stress, damage DNA, and impair gametogenesis [5], [6], [7]. Among the environmental contributors, exposure to heavy metals such as cadmium (Cd), lead (Pb), mercury (Hg), chromium (Cr), arsenic (As), and nickel (Ni) has emerged as a potentially significant but under-recognized risk factor for impaired fertility [8], [9], [10], [11].

Several biological mechanisms may contribute heavy metal-related male reproductive toxicity. Experimental and epidemiological evidence suggests that metal exposure may induce oxidative stress, inflammation, apoptosis, disruption of the blood–testis barrier, and dysfunction of Sertoli and Leydig cells [12]. In addition, it has been shown that these metals may negatively impact the hypothalamic-pituitary axis and impair spermatogenesis, potentially leading to alterations in both semen parameters and circulating reproductive hormones, including testosterone, follicle-stimulating hormone (FSH), luteinizing hormone (LH), and estradiol [10], [13], [14].

Despite the biological mechanistic evidence, several studies investigating the association between heavy metal exposure and reproductive outcomes have demonstrated inconsistent and contradictory results. Previous studies have reported adverse associations of Pb and Cd exposure with male reproductive outcomes, whereas findings for other metals, including As, Hg, Cr, and Ni, have been less consistent. Evidence concerning reproductive hormones is also heterogeneous; associations have been reported for some metals and hormones, but the direction and magnitude of these relationships vary across studie [15], [16], [17], [18].

Given the growing concern over environmental contaminants and reproductive health, it is critical to synthesize the available evidence systematically. A comprehensive understanding of the association between heavy metal exposure and sex hormones and sperm parameters will not only help explain possible mechanisms of reproductive toxicity but also guide preventive and regulatory strategies to reduce exposure risks. This systematic review and meta-analysis aim to evaluate the association between exposure to heavy metals and sex hormones and sperm parameters.

## Methods

In accordance with the Preferred Reporting Items for Systematic Reviews and Meta-Analyses (PRISMA) guidelines [19] All available data on the association of heavy metals and the reproductive system, including sex hormones and semen parameters, were systematically searched and analyzed. The protocol of this study was registered in PROSPERO with the following registration number: CRD420251052421

### Search strategy and data sources

To achieve the expected sensitivity in searching the electronic resources, while observing the technical points of each of them, PubMed (MEDLINE), Web of Science, and SCOPUS were searched until 31 March 2025, as the most complete related electronic resources. Supplementary Table 1 shows the keywords for the search strategy.

### Inclusion and exclusion criteria

The population of interest in the studies was male partners of infertile couples or men undergoing infertility evaluation in infertility clinics. Studies were required to assess heavy metal concentration (lead, mercury, cadmium, arsenic, chromium, and nickel) in body fluids, including urine, blood, and semen.

Studies assessed the relationship between heavy metal concentration and sperm parameters such as sperm concentration, sperm motility, sperm morphology and sperm count, and semen volume in men were included in this study. Moreover, studies reported data on the association between sex hormones, including testosterone, estradiol, LH, FSH, and heavy metal concentration, were also eligible for this study.

Non-human or in vitro studies and review articles, commentaries, case reports, or conference abstracts lacking primary data were excluded. In addition, studies with incomplete or missing data that could not be retrieved through correspondence with the authors were excluded. Studies published before 2000 were also excluded because of their relatively outdated exposure assessment methods and reproductive outcome evaluation standards.

### Quality assessment and data extraction

After the relevancy assessment of titles, abstracts, and full texts, the quality of the included studies was evaluated using the Newcastle–Ottawa Scale (NOS) [20] for observational studies. The detailed results of the quality assessment are presented in Supplementary Table 2. Data extraction was performed using a predefined template covering citation details, publication year, study period, country, study design, population characteristics, sample size, outcomes of interest, and complementary data. All steps, from database searching to data extraction and quality assessment, were conducted independently by two researchers, achieving a high inter-rater agreement.

### Statistical analysis

Meta-analysis was performed only when at least two independent studies reported comparable correlation coefficients for a specific metal–outcome combination. When fewer than two studies were available, findings were summarized narratively and were not included in the quantitative synthesis. The pooled association between heavy metals and the outcomes was assessed using Pearson correlation coefficients. These values were transformed into Fisher’s Z scores using the r-to-z conversion method to calculate the average correlation. Standard errors were computed based on the variance of the Fisher’s Z values. In addition, spearman correlations were converted to Pearson correlation coefficients, using the following formula:

r(Pearson) = 2 ∗ (sin r(Spearman) ∗ π/6)

The chi-square-based Q test and I-squared statistics are used to assess the Between-study heterogeneity. The result of Q test was regarded to be statistically significant at P < 0.1. Due to expected clinical and methodological heterogeneity across studies, the pooled Fisher’s Z were estimated using a random-effect meta-analysis model (using the Restricted Maximum Likelihood method). Publication bias was not evaluated due to the small number of studies included per analysis. The Stata software 18 (StataCorp LP, College Station, Texas, USA), considered for analyses the extracted data.

## Results

### Study selection process

A systematic search yielded 6482 records. After removing duplicates and excluding studies published before 2000, 3644 records remained for screening. Following the title and abstract screening, 150 studies were selected for full-text assessment. Of these, 18 studies met the inclusion criteria, and 6 studies were included in the meta-analysis (Fig. 1).

**Fig. 1:**
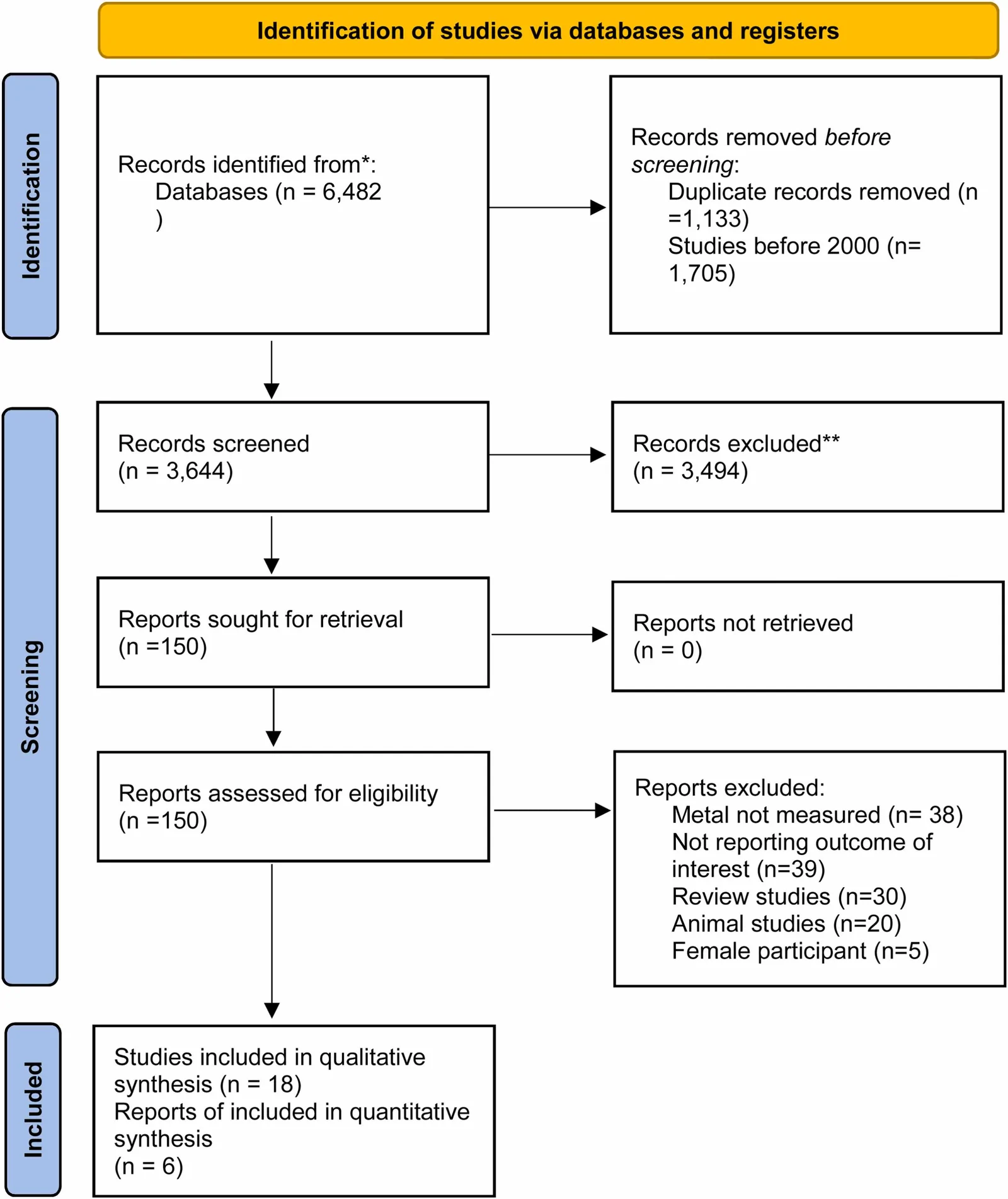
PRISMA flow diagram.

### General characteristics of the included studies

The included studies were published between 2004 and 2021 and involved 4041 participants. Study designs included cohort, case-control, cross-sectional studies, with sample sizes ranging from 42 to 746 participants. Heavy metal concentrations have been assessed in various biological samples, including urine, blood, and semen.

The included studies were conducted across various regions worldwide. Six studies were conducted in China, and three in the USA. Other studies were conducted in Japan, Taiwan, Sri Lanka, Croatia, Tunisia, Poland, Spain, Lebanon, and Nigeria. Table 1 presents the general characteristics of the included studies.

**Table 1:** Characteristics of included studies.

**ID**	**Author**	**Study design**	**Country**	**Sample size**	**Outcome**	**Population**	**Mean/Median age**	**Metal**	**Sampling**	**Measurement**
1	Zhou, L. 2021 [21]	cohort	China	195	SP	men undergoing IVF	31.57 ± 3.62	Cr, Ni, As, Cd, Hg, Pb (μg/L)	Semen	ICP-MS
2	Chabchoub, I. 2021 [22]	case-control	Tunisia	132	SH, SP	men with at least 1 year of occupational exposure to heavy metals	case= 40 (30- 55) control= 38 (31-52)	Cd, Pb(µg/L)	Blood, urine	Shimadzu model AA-6800 AAS
3	Wijesekara, G. U. S.2020 [15]	cross-sectional	Sri Lanka	300	SP	Male partners of infertile couples.	34.83 ± 5.33	Pb μg/dL	Semen	GFAAS
4	Marzec-W*r*óblewska, U. 2019 [23]	cross-sectional	Poland	162	SP	men undergoing infertility evaluation.	NR	Cr, Pb (mg/kg of dry weight)	Semen	ICP-MS
5	Wang, Y. X. 2017 [24]	cross-sectional	China	746	SP	Men from sub-fertile couples.	32 ± 5.1	Cr, As, Cd, Pb (μg/L)	Semen	ICP-MS
6	Wang, Y. X. 2016 [18]	cross-sectional	China	511	SH	Men from sub-fertile couples.	32 ± 5.4	Cr, Ni, As, Cd, Pb (μg/L)	Urine	Agilent 7700x ICP-MS
7	Zeng, Q. 2015 [25]	cross-sectional	China	394	SP	Men sought Semen analysis.	31.4 (22-50)	As, Cd, Cr, Hg, Ni, Pb (μg/g creatinine)	Urine	ICP-MS
8	Zeng, Q. 2013 [26]	cross-sectional	China	118	SH	fertile and sub-fertile men	30.8 ± 5.6	As, Cd, Cr, Hg, Ni, Pb (µg/g creatinin)	Urine	ICP-MS
9	Wu, H. M. 2012 [27]	cross-sectional	Taiwan	341	SP	male partners of infertile couples	34.9 ± 3.7	Pb (μg/l)	Semen	AAS
10	Toshima, H. 2012 [28]	cross-sectional	Japan	42	SP	male partners of infertile couples	36.8 ± 5.4	Cd ng/mL	Urine	ETAAS
11	Li, P. 2012 [29]	cross-sectional	China	49	SP	fertile and infertile men	23–39	As, Pb (ng/mL)	Semen	ICP-MS
12	Mendiola, J. 2011 [17]	case-control	Spain	61	SP, SH	composed of men with oligo-astheno-teratozoospermia and normospermic patients	33.5 ± 3.8	Pb, Cd, Hg (μg/L)	Semen, Blood, Whole blood	ASV and AAS using a Mercury Analyzer.
13	Meeker, J. D. 2010 [30]	Cross-sectional	USA	219	SH	fertile men and men with a range of fertility problems.	34 ± 5.97	As, Cd, Cr, Pb, Hg (μg/L)	Blood	Perkin Elmer ICP-MS
14	Benoff, S. 2009 [31]	Cross-sectional	USA	191	SP, SH	Infertile patients, Sperm donors, and general population	19–55	Cd (μg/L)	Blood, Semen	GFAAS
15	Meeker, J. D 2008 [32]	Cross-sectional	USA	219	SP	Fertile men and men with a range of fertility problems.	34	As, Cd, Cr, Pb, Hg (μg/L)	Blood	Elan DRC Plus ICP-MS
16	Inhorn, M. C. 2008 [33]	case–control	Lebanon	138	SP	men seeking IVF treatment	38	As, Cd, Pb (µg/L)	Blood	7500c ICP-MS
17	Akinloye, O. 2006 [9]	case-control	Nigeria	100	SP, SH	infertile couples with sperm counts less than 20 million/cm3 and healthy fertile controls	20–45	Cd(mg/L)	Blood, Semen	AAS
18	Jurasović, J. 2004 [34]	cross-sectional	Croatia	123	SH	Male partner of an infertile couple with no occupational exposure to metals	19–48	Cd, Pb (µg/L)	Blood	AAS

As, arsenic; Cd, cadmium; Cr, chromium; Hg, mercury; Ni, nickel; Pb, lead; ICP-MS, inductively coupled plasma mass spectrometry; ICP-OES, inductively coupled plasma optical emission spectrometry; AAS, atomic absorption spectrometry; MS, mass spectrometry; GFAAS, graphite furnace atomic absorption spectrometry; HPLC-ICP-MS, high-performance liquid chromatography–inductively coupled plasma mass spectrometry; ETAAS, electrothermal atomic absorption spectrometry; ASV, anodic stripping voltammetry; ICP, inductively coupled plasma, SP: sperm parameters, SH: sex hormones IVF: in vitro fertilisation, NR: not reported.

### Qualitative synthesis of included studies

#### Semen volume

Five studies [9], [15], [23], [27], [32] examined the association between ejaculation volume and trace elements, including As, Cd, Cr, Hg, and Pb. Except for one study [15], which reported a statistically significant negative correlation coefficient between semen volume and Pb (r = -0.25, p-value: 0.007), the remaining studies identified no significant association between ejaculate volume and the trace elements investigated.

#### Sperm concentration and sperm count

##### Arsenic

Among the five studies [21], [24], [25], [29], [32] that examined the relationship between As level and sperm concentration, two reported statistically significant associations. In the study by Li et al. [29] a significant negative correlation was observed between As levels and sperm concentration (r = -0.328, p = 0.021). Similarly, Wang et al. [24] found that men in the highest quartile of seminal plasma arsenic concentration had a 16.58% higher sperm concentration than those in the lowest quartile (95% CI: 1.95, 35.60). In contrast, none of the studies examining total sperm count reported significant associations [24], [25], [33].

##### Cadmium

Seven studie [21], [24], [25], [28], [31], [32] explored Cd exposure in relation to sperm concentration. Only two studies reported significant associations. Toshima et al. [28] found a weak positive correlation (r = 0.316, p = 0.044), while Benoff et al. [31] reported a negative correlation in the infertile population (r = -0.189). The remaining studies, including those that measured Cd levels in semen, blood, or urine, did not support a consistent pattern. For total sperm count, Akinloye et al. [9] reported a significant negative correlation between blood Cd and sperm count (r = -0.32), and Inhorn et al. [33] observed a borderline association with below the reference rate of sperm count (OR = 0.46, 95% CI: 0.21, 1.007). However, the other two studies did not identify statistically significant association [25], [35].

##### Chromium

Zhou et al. [21] identified a negative association between log-transformed semen Cr levels and sperm concentration (β = −0.52, 95% CI: −1.36, 0.33). The other four studies [23], [24], [25], [32], including those analyzing blood and urine samples, yielded non-significant findings. Moreover, studies evaluating total sperm count [24], [25] reported non-significant findings.

##### Mercury

Among four studies [17], [21], [25], [32] evaluating Hg levels, only one established a significant association. Meeker et al. [32] reported an association between blood Hg levels and the odds of having a sperm concentration below the reference values (OR = 0.36, 95% CI: 0.12, 1.03). All other studies reported non-significant associations. Additionally, no significant associations were found for total sperm count [25].

##### Lead

Eight studies [15], [17], [21], [23], [24], [25], [29], [32] investigated the relationship between Pb and sperm concentration. While Li et al. [29] observed a significant negative correlation (r = -0.377), and Zhou et al. [21] found a marginally significant negative association (β = −0.37, 95% CI: −0.82, 0.08), the majority of studies did not find significant results. Regarding sperm count, Wu et al. [27] observed a significant negative beta coefficient (β = −4.44 ± 1.87). Nonetheless, other studies showed no significant effects [24], [25], [33].

##### Nickel

Ni exposure was investigated in a limited number of studies [21], [25]. No statistically significant associations were reported for either sperm concentration or total sperm count. (Fig. 2)

**Fig. 2:**
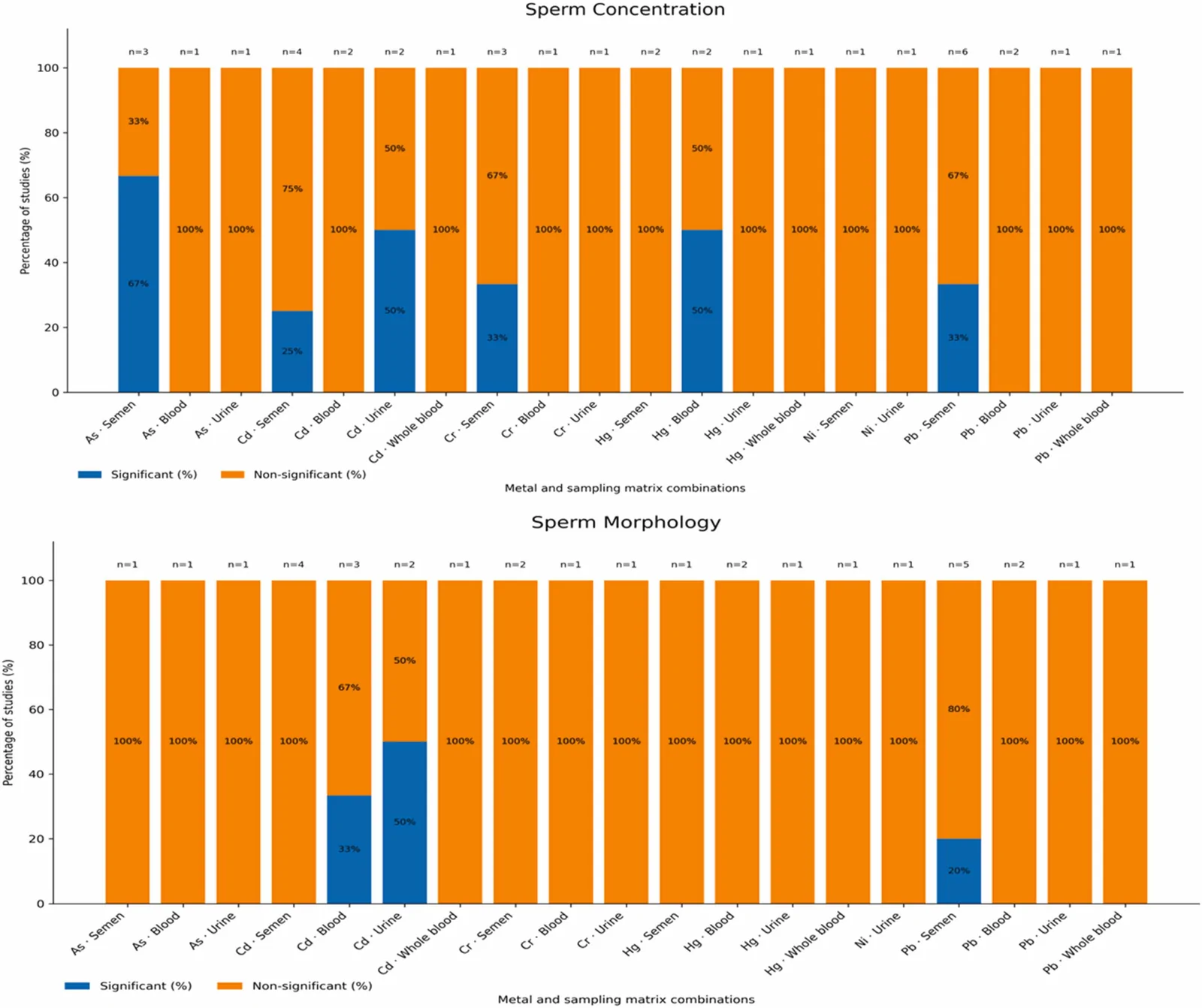
Distribution of statistically significant and non-significant associations between heavy metal concentrations and sperm concentration and sperm morphology.

#### Sperm motility

##### Arsenic

Two studies reported significant inverse associations. Wang et al. [24] observed that higher As concentration in semen (75th vs. 25th percentile in semen) was significantly associated with decreased progressive motility (β = −6.49, 95% CI: −9.82, −2.98) and total motility (β = −6.36, 95% CI: −10.31, −2.57). Meeker et al. [32] reported that higher blood As level was associated with increased odds of impaired sperm motility, compared to men with normal semen analysis. The adjusted odds ratio for the highest exposure category was 3.80 (95% CI: 1.38, 10.4), with a statistically significant trend (p = 0.04). Though the corresponding beta coefficient was non-significant. Other studies found no significant associations for motility across exposure matrices [21], [25], [29], [33] (Fig. 3)

**Fig. 3:**
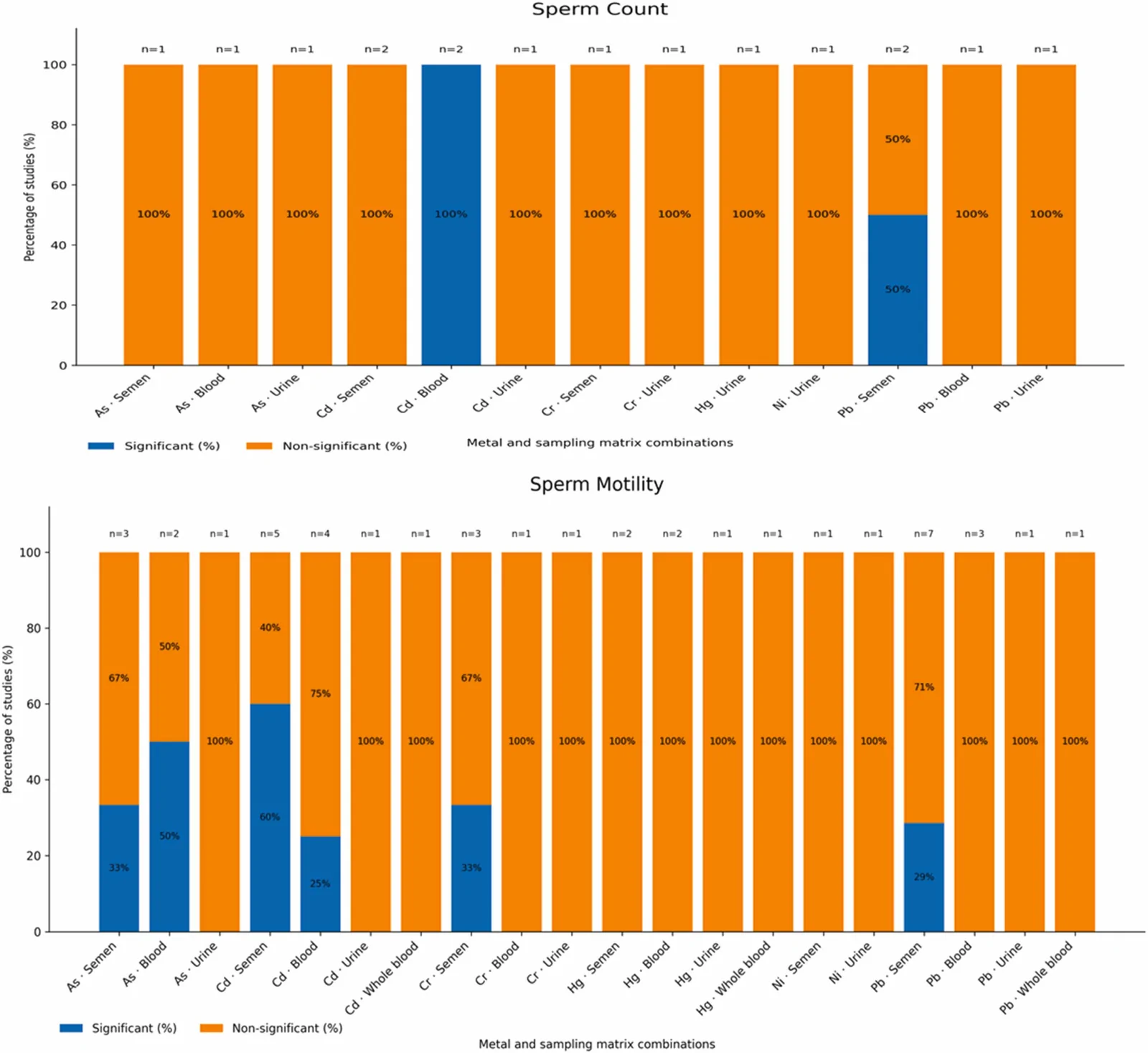
Distribution of statistically significant and non-significant associations between heavy metal concentrations and sperm count and sperm motility.

##### Cadmium

Significant findings were reported by four studies. Wang et al. [24] found that higher semen Cd levels were associated with reduced progressive motility (β = −6.14, 95% CI: −9.82, −2.28) and total motility (β = −5.73, 95% CI: −10.47, −1.63). Mendiola et al. [17] reported a significant association between seminal Cd and increased percentage of immotile sperm (β = 4.9, 95% CI: 0.84, 9.1), whereas no significant relation was found between blood Cd and sperm motility. Akinloye et al. [9] observed strong negative correlations between blood Cd concentrations and both total (r = -0.605) and progressive motility (r = -0.585), while no significant association was observed between sperm motility and seminal plasma Cd. In another study a significant association between seminal Cd and motility was observed (r = –0.201) in infertile group but not in control group. Four other studies did not report significant associations [21], [25], [32], [33] (Fig. 3).

##### Chromium

Five studies evaluated Cr concentrations and sperm motility relations [21], [23], [24], [25], [32]. Zhou et al. [21] found a significant negative association between semen Cr and motile sperm count (β = −0.4, 95% CI: −1.24, 0.45), while other studies found no relation between Cr level and motility.

##### Mercury

No statistically significant associations were reported between Hg concentrations in blood, urine, or semen and sperm motility. [17], [21], [25], [32]

##### Lead

Most studies did not observe significant effects for total or progressive motility [15], [21], [24], [25], [27], [29], [32], [33]. However, Mendiola et al. [17] found that higher seminal Pb were associated with an increased immotile sperm percentage (β = 1.5, 95% CI: 0.37, 1.9), whereas no significant association was observed with blood Pb concentrations. Marzec -W*r*óblewska et al. [23] reported a significant negative correlation (r = -0.2) between immotility and semen Pb along with a weak positive correlation (r = 0.165) with progressive motility.

##### Nickel

Two studies evaluated Ni exposure, none of which reported statistically significant associations with sperm motility. Zhou et al. [21] and Zeng et al. [25], using semen and urine biomarkers respectively, observed effect sizes close to null.

#### Sperm morphology

A total of 10 studies investigated the association between trace elements and sperm morphology [9], [15], [17], [22], [23], [24], [25], [27], [31], [32]. Three studies reported statistically significant associations. Chabchoub et al. [22] found a positive correlation between urinary Cd and abnormal sperm morphology (r = 0.225), whereas Marzec-W*r*óblewska et al. [23] reported a positive correlation between seminal Pb and percentage of normal morphology (r = 0.164). Akinloye et al. [9] observed an inverse correlation between blood Cd concentrations and normal morphology (r = -0.596), while no significant association was observed with seminal plasma Cd concentrations. The remaining seven studies, which evaluated As, Cr, Hg, Ni, and Pb across various biological matrices, did not report statistically significant associations with sperm morphology (Fig. 2).

## Association between heavy metals and sex hormones

### LH and FSH

Among 5 studies investigating the association between trace elements and luteinizing hormone levels [9], [17], [18], [30], [31], only one significant association was found. Meeker et al. [30] found that higher blood As levels (75th vs. 25th percentile) were significantly associated with lower LH levels, with a regression coefficient of β = -0.23 (95% CI: −0.44, −0.02). Similarly, among six studies on FSH [9], [17], [18], [30], [31], [34], only two showed significant associations. Wang et al. [18] reported a 17.84% increase in FSH levels (95% CI: 2.15, 37.23) across the highest vs. lowest quartile of urinary Ni in men. Jurasović et al. [34] also found a positive association with blood Cd (β = 0.194) and a corresponding regression coefficient (B = 2.46, SE = 1.10).

### Testosterone and estradiol

Among eight studies [9], [17], [18], [22], [26], [30], [31], [34] investigating the association between testosterone and trace elements, two reported statistically significant associations (Fig. 4). Jurasović et al. [34], reported a significant positive association between blood Cd concentrations and serum testosterone levels. Similarly, Chabchoub et al. [22] observed a weak positive correlation between blood Pb concentrations and testosterone levels. The remaining six studies, including those on As, Hg, Ni, and Cr, found no significant associations.

**Fig. 4:**
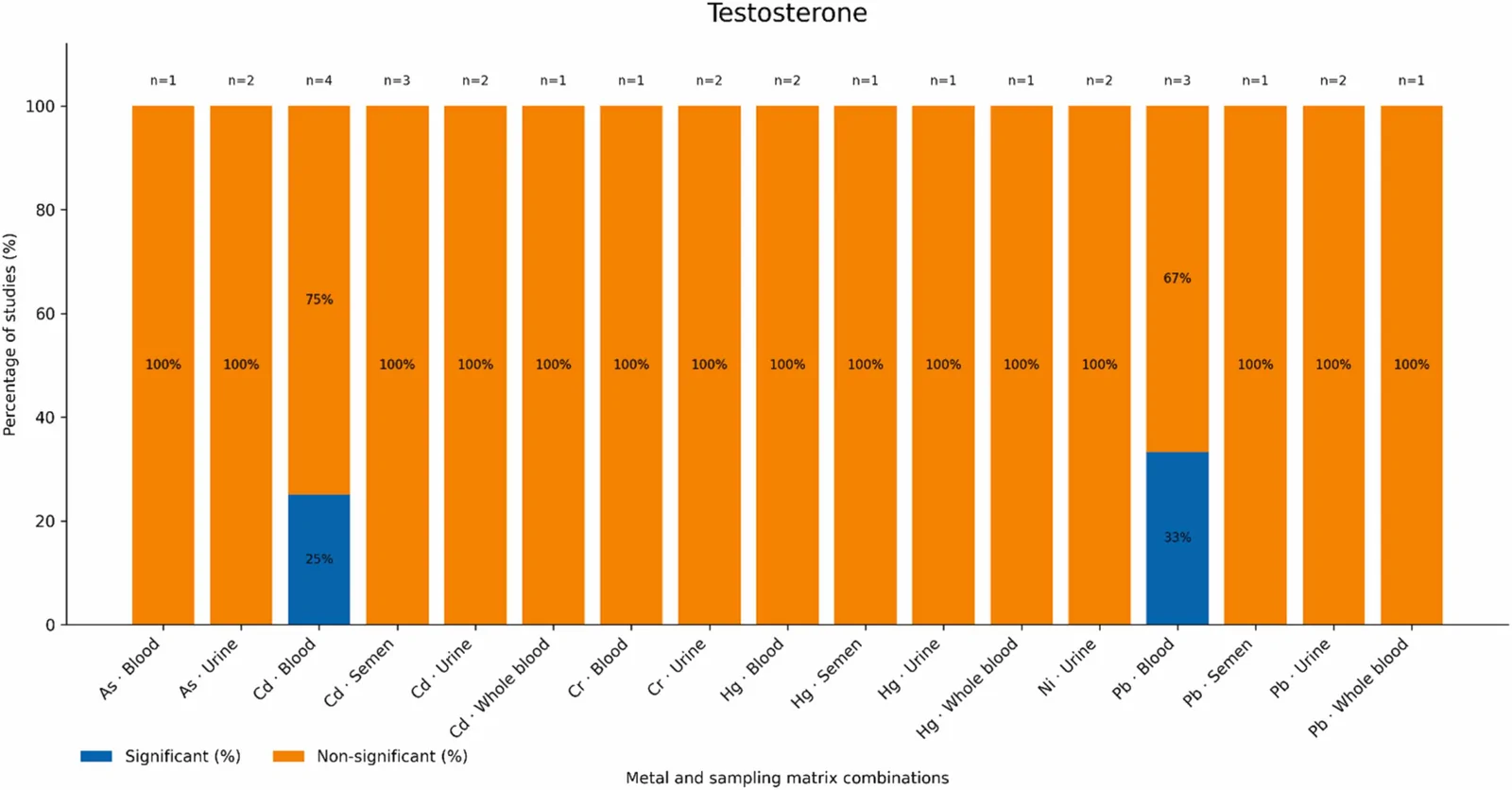
Distribution of statistically significant and non-significant associations between heavy metal concentrations and testosterone.

Two studies investigated the association between trace element concentrations and estradiol levels. Jurasović et al. [34] found a positive association between blood Cd and estradiol, In contrast, Wang et al. [18] did not report significant associations between urinary Cr, Ni, As, Cd, or Pb concentrations and estradiol levels.

Detailed summary of effect sizes and statistical significance for all included studies is provided in Supplementary Table 3.

## Quantitative synthesis (Meta-analysis)

### Pooled association between heavy metals with sperm parameters

1. **Seminal Cd:** Seminal Cd was significantly inversely associated with sperm motility (pooled Fisher’s z = -0.17, 95% CI: −0.30, −0.04; I² = 0%). However, no statistically significant associations were observed for morphology (Table 2).

**Table 2:** Pooled Fisher’s z estimates and heterogeneity statistics for the association between heavy metals and sperm parameters.

**Metal**	**Outcome**	**Study count**	**Pooled result**	**Heterogeneity**			
				**Chi-squared**	**I**^**2**^	**P-value**	**Model**
**Semen**							
**Cd**	Normal morphology	2	-0.07 (-0.22, 0.07)	3.35	11.73	0.34	Random effect
	Sperm motility	3	**-0.17 ( −0.30, −0.04)**	1.32	0	0.70	Random effect
**Pb**	Normal morphology	3	0.04 (-0.10, 0.17)	4.70	58.52	0.10	Random effect
	Progressive motility	2	0.06 (-0.19, 0.30)	4.02	75.14	0.04	Random effect
	Semen volume	3	-0.06 (-0.24, 0.13)	8.35	79.27	0.02	Random effect
	Sperm concentration	3	-0.09 (-0.36, 0.18)	8.81	81.32	0.01	Random effect
	Sperm motility	3	-0.12 (-0.34, 0.09)	6.63	74.65	0.04	Random effect
2. **Seminal Pb:** For seminal Pb, no significant associations were observed with normal morphology, progressive motility, semen volume, sperm concentration, and sperm motility.

Meta-analysis for sex hormones was not possible because fewer than two studies reported comparable effect estimates for any metal-hormone pair.

## Quality assessment

The NOS scores ranged from 6 to 8 across the included studies, with most studies scoring 7 or 8, indicating generally satisfactory methodological quality. Some limitations were observed in specific NOS domains, particularly among the cross-sectional studies; however, no study received an exceptionally low overall score.

## Discussion

This systematic review and meta-analysis synthesized data from 18 studies comprising 4041 participants to examine the relationship between various heavy metals, including As, Cd, Cr, Hg, Ni, and Pb, and reproductive health outcomes in men. The quantitative finding was a significant inverse association between seminal Cd concentrations and sperm motility. In contrast, pooled analyses did not demonstrate significant associations between seminal Pb concentrations and sperm parameters. The review of studies investigating the association between these metals and sex hormone levels revealed inconsistent and contradictory findings.

The inverse association observed between seminal Cd and sperm motility is broadly consistent with previous evidence suggesting adverse association of Cd exposure with male reproductive function. A meta-analysis of 20 studies reported significantly higher concentrations of Pb and Cd in the semen of men with reduced fertility compared to those with normal fertility [36]. Similarly, another meta-analysis of 11 studies found significantly elevated seminal Cd levels among infertile men [37]. In contrast to Cd, the pooled analyses for Pb did not identify statistically significant associations with the evaluated semen parameters. This finding does not necessarily contradict previous meta-analyses reporting higher Pb concentrations among infertile men. Earlier studies often compared Pb concentrations between infertile and fertile groups or evaluated populations with relatively high occupational exposure [36], whereas the present review primarily examined associations between metal and specific reproductive outcomes among men undergoing infertility-related evaluation.

A 2020 meta-analysis highlighted that occupational Pb exposure in males was associated with higher blood Pb levels, reduced sperm count and motility, and increased serum prolactin levels, although no significant differences were found in testosterone, FSH, or luteinizing hormone levels between exposed and unexposed individuals [38]. These differences in exposure intensity, study population, biological matrix, outcome definition, and statistical approach may partly explain the variability in findings across studies.

The current analysis found limited and inconclusive evidence regarding the reproductive impact of As, Cr, Hg, and Ni. However previous studies have suggested that Hg and Cr may adversely affect fertility [39], [40].

The biological plausibility of these associations is supported by mechanistic evidence. Heavy metals are known to induce oxidative stress, damage DNA, and interfere with endocrine signaling pathways, all of which are recognized contributors to reproductive dysfunction [41], [42], [43]. For instance, a study examining Cr toxicity in monkeys reported chromatin fragmentation, mitochondrial swelling, and cytoplasmic vacuolation in spermatocytes, indicating that Cr may impair spermatogenesis through oxidative mechanisms [44]. In parallel, As exposure has been shown to elevate reactive oxygen species (ROS) in the testes of male mice [45] and has been implicated in broader reproductive dysfunction—including reduced sperm quality, hormone disruption, and damage to gonadal tissues in both sexes [46]. Pb exposure is also associated with hormonal alterations, such as elevated testosterone and sex hormone-binding globulin [47]. Furthermore, endocrine-disrupting chemicals (EDCs), including heavy metals, may mimic or interfere with endogenous hormone activity by binding to thyroid hormone receptors (TRs) and sex hormone receptors such as estrogen (ERs), androgen (ARs), and progesterone receptors (PRs), thereby affecting hormonal synthesis, metabolism, and signaling pathways [48], [49], [50]. Pb toxicity has additionally been linked to altered thyroid function, characterized by reduced thyroid-stimulating hormone (TSH) and elevated thyroxine (T4) levels [51]. Experimental studies have also demonstrated that mercury chloride (HgCl₂) exposure leads to reduced sperm quality, lower fertility rates, and decreased offspring survival in male mice [52]. Collectively, the epidemiological and experimental evidence reinforces the endocrine-disrupting potential of heavy metals. However, it is important to note that most animal studies have examined single-metal exposures, which do not fully capture the complexity of combined exposures more reflective of real-world environmental conditions [53].

The evidence regarding reproductive hormones was more limited than that for semen parameters. Although individual studies reported statistically significant associations for selected metal–hormone pairs, including associations involving Cd, Pb, As, and Ni, these findings were not consistently reproduced across studies, biological matrices, or hormone outcomes. This pattern is consistent with previous systematic reviews showing substantial variability in both the direction and magnitude of associations between heavy metal exposure and reproductive hormone levels. Similarly, previous meta-analytic evidence on Pb exposure found no significant differences in testosterone, FSH, or LH despite reported adverse associations with selected semen parameters. Overall, the available evidence does not support a uniform endocrine response to heavy metal exposure [14], [38].

From a public health standpoint, the findings of this review support the continued attention to potentially modifiable environmental and occupational metal exposures in male reproductive health. Assessment of occupational and environmental exposure history may help identify individuals with potentially elevated exposure who could benefit from targeted exposure-reduction counseling or further clinical evaluation. In parallel with the growing emphasis on fertility preservation and assisted reproductive technologies (ART) across diverse populations in recent years [54], [55], [56], [57], clinicians may benefit from systematically investigating potential sources of heavy metal exposure—such as contaminated water or food, residential proximity to industrial sites, and the use of traditional remedies—as part of a comprehensive patient history. Individuals, particularly those in younger age groups, who are exposed to elevated levels of heavy metals may benefit from early reproductive health assessments, tailored counseling aimed at mitigating exposure-related risks, and, where appropriate, consideration of fertility preservation options. In addition, meta-analyses suggest that vitamin E and vitamin D3 may improve some semen parameters in infertile men [58], [59]; however, the evidence is not sufficient to recommend these interventions specifically for heavy metal-related reproductive toxicity.

## Strengths and limitations

The present study has several methodological strengths. It delivers a comprehensive and parameter-specific synthesis of the existing evidence on heavy metal exposure and reproductive function, with separate consideration of metal type, biological matrix, and reproductive endpoint. By simultaneously evaluating semen quality indices and sex hormone profiles, the study captures both direct and endocrine-mediated pathways of reproductive toxicity. Furthermore, the inclusion of globally diverse populations, rather than exclusively occupational or region-specific cohorts, enhances the external validity and generalizability of the findings.

However, several limitations should be considered when interpreting these results. Correlation coefficients are sensitive to the variance and range of exposure within individual study populations.

Consequently, differences in exposure ranges, population characteristics, and exposure assessment methods across studies may have contributed to the observed heterogeneity and should be considered when interpreting the pooled estimates. In addition, the number of studies available for several meta-analyses was limited, which may have reduced the statistical power of some analyses. Moreover, the predominance of cross-sectional studies limits the ability to draw causal inferences. Publication bias could not be formally evaluated because of the small number of studies contributing to each pooled analysis. Consequently, the potential influence of small-study effects cannot be ruled out. Additionally, limited number of studies for As, Cr, Hg, and Ni, prevented conclusion for most of these metal–outcome combinations.

Future research should focus on large-scale prospective cohort studies to better establish causality and temporal relationships. There is also a need to evaluate the combined effects of multiple heavy metals to more accurately reflect real-world exposure scenarios. Mechanistic studies exploring gene–environment interactions may provide further insight into individual susceptibility. Finally, intervention trials assessing the effectiveness of mitigation strategies in reducing metal-related reproductive harm would be valuable for informing public health policy.

## Conclusion

This study suggests that the relationship between heavy metal exposure and reproductive outcomes is parameter-specific rather than universal. While significant inverse associations were observed for seminal cadmium with sperm motility, the overall evidence across sperm parameters and hormone profiles was inconsistent and heterogeneous. These findings highlight the complexity of metal-related reproductive toxicity and suggest that effects may vary according to the type of metal, biological matrix, and specific reproductive endpoint.

## CRediT authorship contribution statement

**Saba Goodarzi:** Writing – review & editing, Writing – original draft, Visualization, Formal analysis, Data curation. **Arman Shafiee:** Writing – review & editing, Methodology, Investigation. **Pegah Rashidian:** Writing – original draft, Data curation. **Reza Ghoreshi:** Writing – review & editing. **Shirin Djalalinia:** Writing – original draft, Methodology. **Zahra Nouri Ghonbalani:** Writing – original draft. **Razieh Lotfi:** Validation, Project administration. **Zohreh Mahmoodi:** Validation, Project administration, Methodology, Conceptualization. **Mostafa Qorbani:** Writing – review & editing, Validation, Project administration, Methodology, Formal analysis, Conceptualization.

## Ethical statement

Not applicable.

## Consent for publication

Not applicable.

## Declaration of Competing Interest

The authors declare that they have no known competing financial interests or personal relationships that could have appeared to influence the work reported in this paper.

## Data Availability

All data has been presented in the manuscript.
